# Whole slide images for primary diagnostics of urinary system pathology: a feasibility study

**DOI:** 10.12861/jrip.2014.26

**Published:** 2014-12-01

**Authors:** Shaimaa Al-Janabi, André Huisman, Geertruida N. Jonges, Fiebo J.W. ten Kate, Roel Goldschmeding, Paul J. van Diest

**Affiliations:** ^1^Department of Pathology, University Medical Center Utrecht, Utrecht, The Netherlands

**Keywords:** Diagnostics, Digital pathology, Urinary pathology, Validation, Whole slide images

## Abstract

**Introduction:** During the last decade, whole slide images (WSI) have been used in many areas of pathology such as teaching, research, digital archiving, teleconsultation and quality assurance testing. However, WSI have as yet not much been used for upfront diagnostics because of the lack of validation studies.

**Objectives:** The aim of this study was to test the feasibility of WSI for primary diagnosis of urinary tract pathology.

**Materials and Methods:** 100 consecutive urinary tract biopsies and resections which had been diagnosed conventionally between the years 2008-2009 were scanned at 20× magnification, and rediagnosed by two pathologists on WSI, having the original clinical information available, but blinded to the original diagnoses. Original and WSI diagnoses were compared and classified as concordant, slightly discordant (without clinical consequences) and discordant.

**Results:** Original and WSI based rediagnosis were concordant in 87% of the cases. Original and WSI diagnosis were slightly discordant in 8% of cases. Major discrepancies with clinical or prognostic implications were founded in only 5 cases. However, for 6 out of the 13 discrepancies, WSI based diagnoses were considered to be better than the original diagnoses.

**Conclusion:** Primary diagnostics of urinary tract specimens can be reliably done on WSI. Further improvements of image resolution may help to increase diagnostic accuracy and WSI acceptance in routine pathology.

**Implication for health policy/practice/research/medical education:**

Primary diagnostics of urinary tract pathology can be reliably done on whole slide images. Further improvements of image resolution may help to increase diagnostic accuracy and whole slide images acceptance in routine pathology.

## Introduction

Whole slide imaging technology allows automatic digitization of the entire glass slides, producing what is commonly referred to as digital slides or whole slides images (WSI). WSI are usually examined on a computer screen by the aid of image viewers enabling the examination and manipulation of the whole tissue section in a way comparable to a conventional microscope.

Easy image annotation, accessibility, sharing as well as the possibility of capturing static images for documentation, insertion of comments, and subjection of automated image analysis are all additional features intimately bound to WSI making them superior to using glass slides and a conventional microscope for several applications within the pathology workflow. Simultaneous viewing of WSI by different examiners from different places makes WSI more suitable for education, teleconsultation, pathology panels and revision. WSI can be digitally archived and retrieved minimizing the time and effort needed for preparing slides for revision or conferences. Moreover, the possibility of linking WSI to a patient’s complete medical record could increase the diagnostic accuracy and decrease the errors resulting from the lack of clinically relevant information. To this end, linking WSI and patients’ information to a central storage will facilitate teleconsultation and telerevision resulting in enhancing patients’ care.

Despite all the advantages of digital pathology and WSI, unfortunately their use as a tool for primary diagnostics is still not widespread. One of the factors hindering WSI integration in routine pathology practice is that they have not yet been approved for primary diagnostics by the food and drug administration (FDA) in the USA and the scanners to acquire WSI have been classified as class III medical devices. This makes the approval process very time consuming and expensive for scanner vendors. One of the required steps will be to setup collaborations with multiple pathology laboratories for large scale multicenter validation studies aimed for systematic validation of WSI for primary diagnostic purposes.

## Objectives

The aim of this study was to evaluate the feasibility of primary pathology diagnosis of urinary system specimens using WSI by comparing this to the performance when using a conventional microscopy. This study is a part of a larger study aimed for systematic validation of WSI for primary diagnostics in a number of different organ systems (1-4).

## Materials and Methods

For this study 100 cases (50 from kidney and 50 cases from other parts of the urinary system) with a complete set of well focused WSI that had been conventionally diagnosed by two pathologists in 2008-2009 were selected. The same pathologists who did the initial diagnosis were asked to re-diagnose their own cases on WSI to exclude inter-observer variation as much as possible. The time period between the primary microscopic diagnosis and re-diagnosis on WSI ranged from 6 months to one year to guarantee wash out. The participating pathologists had varying but at least 1 year experience in using WSI for secondary diagnostics (tumor boards, education, reviewing archived slides, etc.).

WSI were per case presented to the pathologists together with the original clinical information, blinded to the original report. The selected cases consisted of 89 biopsies and 11 resections from kidney and other parts of the urinary system. Table 1  summarizes these cases in relation to their origin and the type of the specimen (biopsy or resection). Table 2  and 3  detail the diagnostic entities of cases included in this study.

**Table 1:** Specimen type and origin in the urinary tract of cases included in this study

**Organ**	**Specimen type**		**Total**
	**Biopsy**	**Resection**	
Kidney	45	5	50
Bladder	41	2	43
Ureter		1	1
Urethra	3	3	6
Total	89	11	100

**Table 2:** Primary diagnoses of the fifty cases originating from the kidney

**Disease category**	**Kidney type**		**Total**
	**Native**	**Transplant**	
Vascular		6	6
Glomerular	11	2	13
Tubulointerstitial	2	13	15
Tubulointerstitial and vascular		6	6
Tubulointerstitial, vascular and glomerular		1	1
Developmental anomaly	2		2
No specific abnormality		5	5
Carcinoma	2		2
Total	17	33	50

**Table 3:** Primary diagnoses of the fifty non-kidney cases

**Location**	**Diagnosis entity**			**Total**
	**Benign**	**Neoplastic**	**Normal**	
Bladder	22	16	5	4 3
Ureter	1			1
Urethra	4	2		6
Total	27	18	5	50

The original and WSI based diagnoses were compared by three independent pathologists to judge the concordance between the two diagnoses as before (1-4) as:

**Table 4:** Details of all cases with discrepancies between light microscopic and digital diagnoses.

**No**	**Tissue** **type**	**Microscopic diagnosis**	**Digital diagnosis**	**Discrepancy type**	**Preferred diagnosis**
1	TK	Acute cellular tubulointerstitial rejection and suspicion of vascular rejection	Chronic damage with reactive inflammatory infiltrate. Insufficient evidence for acute rejection or toxicity	Discrepant	Both imperfect
2	TK	Acute vascular rejection (Banff IIA) with thrombotic microangiopathy	(Sub-)acute thrombotic microangiopathy	Discrepant	Original
3	TK	Kidney biopsy with an acute borderline cellular tubulointerstitial rejection	Less than 5 % IFTA, slight ischemic changes in the glomeruli. Insufficient evidence for rejection	Discrepant	Original
4	TK	Slight acute tubular damage. No signs of rejection or ATN	Calcineurin inhibitor toxicity. Insufficient evidence for rejection	Discrepant	Digital
5	TK	Acute borderline cellular tubulointerstitial rejection	Calcineurin inhibitor toxicity. Insufficient evidence for rejection	Discrepant	Digital
6	TK	Biopsy with heavy inflammation and signs of acute tubulointerstitial rejection (Banff grade IA) BK –negative	Severe acute tubulointerstitial rejection, with heavy inflammatory infiltrate with apparent disruption of tubular basement membrane, suggestive of Banff grade 1B acute tubulointerstitial rejection	Mildly discrepant	Original
7	TK	Granulomatous TIN. Drug induced? Acute cellular tubulointerstitial rejection cannot be excluded.	Antibody mediated rejection (capillaritis). Acute cellular tubulointerstitial rejection with Granulomatous reaction and destruction of tubules consistent with Banff grade IB rejection	Mildly discrepant	Digital
8	TK	Tubulointerstitial and vascular rejection (Banff grade IIA) Suspected antibody mediated rejection component	Tubulointerstitial rejection (Banff grade IA)	Mildly discrepant	Digital
9	Bladder	TUR with small location of transitional cell carcinoma grade 3 without evidence of invasive growth in addition to the presence of loose group of cells which is strongly atypical	Grade 3 transitional cell carcinoma, invasive in lamina propria	Mildly discrepant	Both imperfect
10	Bladder	Erosive active chronic inflammation with the presence of loose atypical tissue fragments which cannot be good assessed	Necrosis and moderate chronic inflammation, insufficient for CIS diagnosis	Mildly discrepant	Original
11	Bladder	Mechanical tissue damage with papillary transitional cell carcinoma grade 3. The picture is suspicious for superficial invasive growth but no definite diagnosis	Papillary transitional carcinoma grade 3, focally invasive in lamina propria with well circumscribed CIS	Mildly discrepant	Original
12	Bladder	Large fragment of muscular tissue without malignancy with the presence of superficial fragments of transitional cell carcinoma, no invasion	Loose tumour cells (transitional cell carcinoma) and muscles fragments. Invasion cannot be assessed	Mildly discrepant	Digital
13	Bladder	Bladder biopsy without specific abnormality	Chronic inflammation	Mildly discrepant	Digital

TK: transplanted kidney, TUR: Transurethral resection, TIN: Tubulointerstitial nephritis, ATN: Acute tubular necrosis, BK: virus, CIS: carcinoma in situ, IFTA: interstitial fibrosis and tubular atrophy

- Concordant; complete agreement between the first original signed out diagnosis and the diagnosis as drawn from the whole slide image
- Slightly discrepant; mild differences which would not have any clinical or prognostic implications
- Discrepant; differences with clinical and/or prognostic implications for the patient

The better one of the two diagnoses (original or WSI based) was noted.

## Results

For 87 out of 100 cases (87%, 95% CI 0.80-0.94), the light microscopy and the WSI based diagnosis were concordant. Of the other 13 cases, eight showed slight discordance between the digital and the light microscopic diagnoses without any clinical or prognostic implications for the patient, while in five cases the discrepancy could have an effect on patient treatment and prognosis.

Re-assessment of the glass slides and the WSI for the discrepant cases by the three reviewing pathologists revealed that in six cases the WSI diagnosis was preferred over light microscopy diagnosis while the original light microscopy based diagnosis was preferred in five cases. However, in two cases (one discrepant and one slightly discrepant) both diagnoses gave imperfect description to the problem. Table 4  details these discrepant cases.

In the subgroup of 50 biopsies and resections that originated from the kidney, the original microscopic diagnoses were concordant with WSI based diagnoses in 42 cases (84%, 95% CI: 0.73-0.95). In five cases, there were major discrepancies with possible clinical implications on the patient treatment and prognosis. Reassessment of the glass slides for these discrepant cases by the reviewing pathologists revealed that WSI based diagnoses were preferred in two discrepant cases and the original light microscopic diagnoses were preferred for two cases as well. For one discrepant case, both digital and light microscopic diagnoses gave imperfect description of the underlying pathology. In this case, tubulointerstitial rejection and suspicion of vascular rejection had been stated microscopically which was not confirmed digitally (case 1, Table 4 ). On revision by conventional microscopy, the presence of tubulointerstitial rejection was confirmed but evidence of vascular rejection was considered insufficient.

For renal specimens, discrepancies were mostly related to over- or underestimation of rejection. In addition, there were about 3 other mildly discrepant cases where the difference between conventional microscopy and WSI would not have an effect on patient treatment and prognosis.

In the subgroup of 50 cases that originated from the other parts of urinary system, the WSI based diagnoses were concordant with the light microscopic diagnoses in 90% of the cases (95% CI: 0.81-0.99). All of these discrepancies were mild without further clinical implication. Reassessment of the glass slides by the reviewing pathologists, revealed that the WSI based diagnoses were preferred in two cases and the original light microscopic diagnoses were preferred again in two cases, whereas in one case both diagnoses were imperfect (case 9, Table 4 ). In this resection, invasion was proposed digitally but could not be confirmed microscopically. On revision, both diagnoses were considered to be imperfect and “invasion cannot be excluded” was concluded to be the best description of the lesion.

## Discussion

The aim of this study was to test the feasibility of using WSI for primary diagnosis of tissue biopsies and resection specimens originating from the kidney and other parts of the urinary tract. One hundred cases received between 2008 and 2009 were retrospectively collected and blindly re-diagnosed by two pathologists on the bases of WSI after a wash-out period of at least six months. The re-diagnosis was done by the same pathologists who performed the initial diagnosis to avoid inter-observer variations due to e.g. difference in experience. The re-diagnoses were concordant with the original light microscopy diagnosis in 87% of cases (95% CI 0.80-0.94). There were mild discrepancies between the light microscopy and the WSI based diagnoses in 8% of the cases, without clinical or prognostic implications to the patients. However, in 5 cases (5%) the pathology reports obtained by the two diagnostic modalities were discrepant with potential impact on the patient’s treatment.

The concordance rate of 87% and the mild rate of discrepancies are within the range of previously observed inter- and intra-observer variability in microscopic pathology in general (5-7) and in renal pathology specifically (8-13), and is in line with previous similar studies by us in other organ systems (1-4). Furthermore, in 6 out of 13 discrepancies the WSI diagnoses were deemed better. These results indicate that WSI may reliably be used for primary diagnostics of urinary system specimens.

Despite the fact that several studies have emphasized the benefits of WSI in different pathology applications and also in primary diagnostics, integrating WSI in the routine workflow will probably not be achieved unless pathologists are convinced that the diagnostic performance on WSI is not inferior to a light microscopy based diagnoses based on glass slides (14). This requires solid evidence obtained from well-designed validation studies genuinely reflecting the reliability of digital pathology.

WSI based diagnostics offers a seamless and reliable medium for revising cases and providing pathology diagnostic services especially to remote hospitals lacking an on-site pathologist. This fact has been illustrated in a study of Furness *et al*. where the adequacy of WSI as a medium for internet-based telepathology was evaluated by multiple examiners in the context of The National Renal Pathology External Quality Assurance scheme in the UK (15). Their results have shown no significant difference between the diagnostic accuracy of the pathology reports derived from WSI and conventional microscopy; this could endorse the frequent use of this technology in the quality assurance programs.

The results in the present study are comparable to other studies that evaluated the validity of WSI for primary diagnostics of renal specimens. In a study by Ozluk *et al*., three pathologists scored 11 pathologic criteria derived from the Banff classification of renal transplant in 40 renal biopsies and eventually constructed the final conclusion of acute rejection or transplant glomerulopathy. Each biopsy was examined by each observer independently on four occasions; twice microscopically and twice on WSI with at least 3 weeks time in between each diagnostic modality. Their results revealed good intraobserver reproducibility of Banff scoring system using WSI as well as glass slides. Moreover, there was no significant difference in evaluating acute rejection using both diagnostic methods. Glomerulopathy scoring was the most reproducible feature with almost similar accuracy between glass slides and WSI (16). The drawback of this study is the multiple readings within a relatively short time, as the pathologists might have remembered the diagnosis in some of the cases.

Jen *et al*. investigated the validity of WSI in evaluating renal allograft biopsies. Six pathologists assessed the presence of certain morphologic features and acute rejection in 25 renal biopsies using conventional microscope and WSI with at least a period of two weeks in between the two diagnostics. Their results showed substantial agreement between glass slides and WSI based diagnoses in assessing specific morphologic criteria and acute rejection. Moreover, the inter-observer agreement was shown to be comparable between the two diagnostic modalities (17). The low number of cases included in that study and the short time between the examinations of the cases are however limitations of their study.

The resolution of WSI scanned at 20x was perceived to be on the low side for rendering diagnostics of renal specimens. Evaluating the status of transplanted kidney and the possibility of transplant rejection requires careful assessment of fine morphologic features among which is the presence of inflammation, in particular tubulitis, fibrosis and subtle changes in glomeruli, blood vessels and tubules. This task was found to be slightly more difficult and time consuming on 20x WSI than in conventional microscopy. Moreover, with the digital readings in this study, clinical information provided on transplant biopsies was generally less extensive than with the original microscopic evaluation, and also feedback from multidisciplinary discussion was lacking, which all might have contributed to discrepancies in 5 cases when comparing digital with conventional readings. However, issues related to lower resolution scan are expected to be solved in the near future especially with the presence of high throughput scanners which are able to scan the whole slide at high resolution and in less than one minute.

Rendering diagnosis on 20x WSI for biopsies and resections from the other parts of the urinary tract was considered to be relatively easier. This was reflected by the higher concordance rate of 90% and the mild discrepancies with minimal clinical impact on patient.

One of the limitations hindering the use of WSI for primary diagnostics of urinary system specimens is the time needed for image exploration. Examining WSI was perceived to take considerably more time than evaluation by conventional microscope (although no formal timing has been conducted). This has also been noted in the study of Jen *et al*. where exploring WSI cost 1.4 longer time than using glass slides and conventional microscope. Relative lack of routine, limited image resolution and suboptimal navigation tools might all have contributed to this difference. We expect that the impact of time factor will be reduced when a high resolution scan becomes a common standard in pathology and with the introduction of more user-friendly interfaces where exploring WSI can be done in simple intuitive way as using efficient tools for navigating through the image instead of the mouse (18).

Implementing WSI in primary diagnostics will enhance pathology practice especially for sub-specialties such as transplantation pathology. With the aid of WSI, problematic or difficult cases can be efficiently shared immediately with one or more experts within suitable time constrains sparing the time required for shipping glass slides to far places. Integrating WSI into a patient’s medical report will allow the pathologists to work within an integral environment including the clinical information, pathology data besides the pathology specimens which will eventually permit comparing new and old patient’s materials to evaluate the progress in the patient’s condition. WSI can also be electronically archived and retrieved decreasing the time spent on searching for glass slides for consultation, conferences, teaching and research purposes. Furthermore WSI can be subjected to automated image analysis which is believed to improve the productivity and objectivity in daily diagnostics.

The above mentioned features may encourage considering WSI as platform for primary diagnostics in pathology. Nevertheless, integrating WSI in routine practice may still require investing the efforts for step-wise conversion from conventional to digital practice.

## Conclusion

Primary diagnostics of urinary tract specimens can be reliably done on whole slide images.

However high resolution scans may be required especially in assessing the status of renal transplants. Thus further improvements of image resolution may help to increase diagnostic accuracy and WSI acceptance in routine pathology.

## Authors’ contributions

Conceived and designed the experiment: SAJ, AH, PJvD. Performed the experiment: SAJ, AH, PJvD, RG, GNJ, FJWtK. Primary formulation of the manuscript: SAJ. This manuscript has been reviewed and edited by the participating authors.

## Conflict of interests

The author declared no competing interests.

## Ethical considerations

Ethical issues (including plagiarism, data fabrication, double publication) have been completely observed by the authors.

## Funding/Support

There is no financial interests related to the material in the manuscript.

## References

[1] Al Janabi S, Huisman A, Vink A, Leguit RJ, Offerhaus GJ, ten Kate FJ (2012). Whole slide images for primary diagnostics of gastrointestinal tract pathology: a feasibility study. Hum Pathol.

[2] Al Janabi S, Huisman A, Vink A, Leguit RJ, Offerhaus GJ, Ten Kate FJ (2012). Whole slide images for primary diagnostics in dermatopathology: a feasibility study et alWhole slide images for primary diagnostics in dermatopathology: a feasibility study. J Clin Pathol.

[3] Al-Janabi S, Huisman A, Nikkels PG, Ten Kate FJ, van Diest PJ (2012). Whole slide images for primary diagnostics of paediatric pathology specimens: a feasibility study. J Clin Pathol.

[4] Al-Janabi S, Huisman A, Willems SM, van Diest PJ (2012). Digital slide images for primary diagnostics in breast pathology: a feasibility study. Hum Pathol.

[5] Fadare O, Parkash V, Dupont WD, Acs G, Atkins KA, Irving JA (2012). The diagnosis of endometrial carcinomas with clear cells by gynecologic pathologists: an assessment of interobserver variability and associated morphologic features. Am J Surg Pathol.

[6] Manchanda A, Shetty DC (2012). Reproducibility of grading systems in oral epithelial dysplasia. Med Oral Patol Oral Cir Bucal.

[7] Costa e Silva IT, Araújo JR, Andrade RV, Cabral CR, Gimenez FS, Guimarães AG (2011). Interobserver variability in the diagnosis of anal cancer precursor lesions: study of the usual scenario. Rev Col Bras Cir.

[8] Joh K, Morozumi K, Kitamura H (2006). Symposium: evaluating the reproducibility of pathological diagnosis using the 1997 Banff classification update. Clin Transplant.

[9] Brazdziute E, Laurinavicius A (2011). Digital pathology evaluation of complement C4d component deposition in the kidney allograft biopsies is a useful tool to improve reproducibility of the scoring. Diagn Pathol.

[10] Veronese FV, Manfro RC, Roman FR, Edelweiss MI, Rush DN, Dancea S (2005). Reproducibility of the Banff classification in subclinical kidney transplant rejection. Clin Transplant.

[11] Sar A, Worawichawong S, Benediktsson H, Zhang J, Yilmaz S, Trpkov K (2011). Interobserver agreement for Polyomavirus nephropathy grading in renal allografts using the working proposal from the 10th Banff Conference on Allograft Pathology. Hum Pathol.

[12] Furness PN, Taub N (2001). International variation in the interpretation of renal transplant biopsies: report of the CERTPAP Project. Kidney Int.

[13] Furness PN, Taub N (2006). Interobserver reproducibility and application of the ISN/RPS classification of lupus nephritis-a UK-wide study. Am J Surg Pathol.

[14] Ghaznavi F, Evans A, Madabhushi A, Feldman M (2013). Digital Imaging in Pathology: Whole-Slide Imaging and Beyond. Annu Rev Pathol.

[15] Furness P (2007). A randomized controlled trial of the diagnostic accuracy of internet-based telepathology compared with conventional microscopy. Histopathology.

[16] Ozluk Y, Blanco PL, Mengel M, Solez K, Halloran PF, Sis B (2012). Superiority of virtual microscopy versus light microscopy in transplantation pathology. Clin Transplant.

[17] Jen KY, Olson JL, Brodsky S, Zhou XJ, Nadasdy T, Laszik ZG (2013). Reliability of whole slide images as a diagnostic modality for renal allograft biopsies. Hum Pathol.

[18] Wang Y, Williamson KE, Kelly PJ, James JA, Hamilton PW (2012). SurfaceSlide: a multitouch digital pathology platform. PLoS One.

